# Supplementary material for: The AENEAS Project: Intraoperative Anatomical Guidance Through Real-Time Landmark Detection Using Machine Vision
Source: Mayo Clin Proc Digit Health. 2025 Dec 1;4(1):100308. doi: 10.1016/j.mcpdig.2025.100308 (PMC12885630; doi:10.1016/j.mcpdig.2025.100308)
Supplement: Supplemental Material [file mmc1.pdf]

Hyperparameters:

lr0: 0.01
lrf: 0.1
momentum: 0.937
weight_decay: 0.0005
warmup_epochs: 3.0
warmup_momentum: 0.8
warmup_bias_lr: 0.1
box: 0.05
cls: 0.3
cls_pw: 1.0
obj: 0.7
obj_pw: 1.0
iou_t: 0.2
anchor_t: 4.0
fl_gamma: 0.0
hsv_h: 0.015
hsv_s: 0.7
hsv_v: 0.4
degrees: 0.0
translate: 0.2
scale: 0.9
shear: 0.0
perspective: 0.0
flipud: 0.0
fliplr: 0.5
mosaic: 1.0
mixup: 0.15
copy_paste: 0.0
paste_in: 0.15
loss_ota: 1

Options:

weights: yolov7x_training.pt
cfg: ''
data:
hyp:
epochs: 300
batch_size: 8
img_size:
- 1920
- 1920
rect: false
resume: false
nosave: false
notest: false
noautoanchor: false
evolve: false
bucket: ''
cache_images: false
image_weights: false
device: 0,1
multi_scale: false
single_cls: false
adam: false
sync_bn: false
local_rank: -1
workers: 8
project:
entity: null
name: exp
exist_ok: false
quad: false
linear_lr: false
label_smoothing: 0.0
upload_dataset: false
bbox_interval: -1
save_period: -1
artifact_alias: latest
freeze:
- 0
v5_metric: false
world_size: 1
global_rank: -1
save_dir:
total_batch_size: 8
